# Blood biochemistry and haematology of migrating loggerhead turtles (*Caretta caretta*) in the Northwest Atlantic: reference intervals and intra-population comparisons

**DOI:** 10.1093/conphys/coy079

**Published:** 2019-02-07

**Authors:** Tiffany Yang, Heather L Haas, Samir Patel, Ronald Smolowitz, Michael C James, Amanda S Williard

**Affiliations:** 1 Department of Biology and Marine Biology, University of North Carolina Wilmington, Wilmington, NC, USA; 2 National Oceanographic and Atmospheric Administration, Northeast Fisheries Science Center, Woods Hole, MA, USA; 3 Coonamessett Farm Foundation, East Falmouth, MA, USA; 4 Fisheries and Oceans Canada, Population Ecology Division, Bedford Institute of Oceanography, Dartmouth, NS, Canada

**Keywords:** Reference intervals, health status, physiology, migration, sea turtle

## Abstract

We documented blood biochemistry and haematology of healthy loggerhead turtles (*Caretta caretta*) in the Northwest (NW) Atlantic in order to establish clinical reference intervals (RIs) for this threatened population. Blood samples were analysed from migratory loggerheads captured off the Mid-Atlantic coast of the USA in 2011, 2012, 2013 and 2016 as part of a long-term research program. Blood variables were determined using a point-of-care analyser, and a veterinary diagnostic laboratory service. We calculated 95% RIs with associated 90% confidence intervals (CIs) for each blood variable. We compared results obtained from our study of migratory loggerheads with published data for similarly sized loggerheads resident at a seasonal temperate latitude foraging area. Significant differences in several blood variables between migratory and resident turtles provided insight on energetic and health status during different behavioural states. Temperature was significantly correlated with several blood variables: lactate, pCO_2_, sodium, haemoglobin and lactate dehydrogenase. Our assessment of blood chemistry in healthy loggerhead turtles in the NW Atlantic provides a baseline for clinical comparisons with turtles impacted by anthropogenic and environmental threats, and highlights the importance of identifying unique aspects of biochemical and haematological profiles for sea turtles at the intra-population level.

## Teaser

We established reference intervals for blood biochemistry and haematology of loggerhead turtles captured off the Mid-Atlantic coast of the USA. This assessment of blood variables in healthy, wild loggerhead turtles allows for comparisons with turtles impacted by anthropogenic and environmental threats, as well as turtles sampled in different habitats and life stages.

## Introduction

Establishing baseline blood biochemistry and haematology profiles, often in the form of reference intervals (RIs), is a common practice for evaluating the clinical health status of wild animals (Bolten and Bjorndal, 1992; Troiano *et al.*, 1997; Samour *et al.*, 1998; Christopher *et al.*, 1999; Stamper *et al.*, 2005; Hidalgo-Vila *et al.*, 2007; Deem *et al.*, 2009; Gelli *et al.*, 2009; Delgado *et al.*, 2011; Basile *et al.*, 2012; Fazio *et al.*, 2012; Lewbart *et al.*, 2014; Muñoz-Pérez *et al.*, 2017). As with human medicine, in veterinary diagnostic laboratories, RIs are typically established as the central 95% of the reference population with 90% confidence limits (CIs), thus creating a narrow range of expectations for clinically healthy animals (Lumsden and Mullen, 1978). RIs provide a clinical baseline that is useful for monitoring health trends in wild populations. For example, Christopher *et al.* (1999) documented biochemical and haematological RIs for desert tortoises which provided a means for analysing differences between sexes, distinguishing seasonal influences on physiological condition, and assessing differences in foraging behaviour between tortoises at three geographic locations. Establishment of RIs also permits assessment of compromised health status due to anthropogenic or environmental disturbances (Kelly *et al.*, 2015). Stacy *et al.* (2017) utilized previously established RIs and expert clinician-based assessments to characterize the health status of marine turtles impacted by the BP Deep Water Horizon oil spill. Physiological status of oiled turtles was monitored by documenting blood biochemistry and haematology throughout the rehabilitation period to assess the full breadth of impact of crude oil exposure and the likelihood of full recovery. Studies such as this provide insight on health problems that may occur in response to anthropogenic or environmental disturbances, and help clinicians and conservation managers provide well-informed response efforts for impacted animals (Stacy *et al.*, 2017).

Our study focused on establishing RIs for the Northwest (NW) Atlantic Distinct Population Segment (DPS) of loggerhead turtles (*Caretta caretta*), which is comprised of loggerhead turtles that inhabit waters on the eastern coast of the USA and Canada (Conant *et al.*, 2009; Wallace *et al.*, 2010). The NW Atlantic DPS is listed as threatened by the US Endangered Species Act (Conant *et al.*, 2009) and endangered by the Canadian Species At Risk Act (Government of Canada, 2017).

This population faces a number of threats such as fisheries bycatch (Brazner and McMillan, 2008; Haas, 2010; Murray, 2011; Murray and Orphanides, 2013), oil and gas explorations (Klima *et al.*, 1988; Bolten *et al.*, 2011), and climate change (Hawkes *et al.*, 2007; Chaloupka *et al.*, 2008). Fisheries interactions, in particular, have been highlighted as a potential source of mortality for loggerheads (Bolten *et al.*, 2011). Even if turtles do not die as a direct result of entanglement or hooking in fishing gear, injuries sustained as a result of capture may result in sublethal impacts that could affect post-release behaviour and fitness (Lewison *et al.*, 2004; Wilson *et al.*, 2014). Previous studies have illustrated variation in blood chemistry between hand-caught and fisheries-caught loggerheads indicative of induction of a stress response and metabolic disturbances (Williard *et al.*, 2015), however, additional data on natural variation in blood variables for healthy turtles are needed in order to appropriately assess impacts of fisheries interactions and other disturbances. Establishment of RIs for loggerhead turtles in the NW Atlantic DPS permits differentiation between healthy and unhealthy turtles and allows for clinically-based, comprehensive assessment and management of populations (Flint *et al.*, 2010a).

A small number of studies have provided biochemical and haematological RIs for NW Atlantic DPS loggerhead turtles in seasonal nearshore foraging habitats along the southeastern coast of the USA (Deem *et al.*, 2009; Kelly *et al.*, 2015). The primary goal of our research was to provide biochemical and haematological RIs for NW Atlantic loggerhead turtles during seasonal migrations in offshore habitats of the US Mid-Atlantic Bight (MAB) (Winton *et al.*, 2018). The physiological status of marine turtles during migration at temperate latitudes may differ from that of turtles residing at lower latitude foraging grounds. Not only do migratory turtles experience high metabolic demands from continual swimming (Papi *et al.*, 1997; Bowen *et al.*, 2005), but the energetic demands of migration may occur in tandem with shifts in behaviour and environmental factors (Solow *et al.*, 2002). Migrating loggerhead turtles exhibit a greater number of shorter duration dives compared with turtles at foraging grounds (Papi *et al.*, 1997), which may reflect a decrease in food intake during directed long-distance movements. Furthermore, as poikilothermic animals, loggerhead turtle behaviour and metabolic function are impacted by the cooler water temperatures experienced at higher latitudes (Mrosovsky, 1980; Davenport, 1997). The creation of biochemical and haematological RIs for loggerhead turtles migrating through offshore habitats in the MAB provides a baseline for clinical health assessments and evaluation of physiological impacts of environmental disturbance, as well as a basis of comparison for the physiology of different behavioural states. It is widely recognized that establishment of blood chemistry RIs at the inter-population level for a given species is necessary in order to account for unique genetics, variety of habitats encountered, and differences in behaviour (Hrubec *et al.*, 2000; Flint *et al.*, 2010b). Establishment of RIs at the intra-population level is warranted given the physiological adjustments that may occur while foraging in nearshore neritic habitats, migrating in pelagic waters, or nesting on land (Prange, 1976; Deem *et al.*, 2009).

The goals for our study were two-fold: (1) establish RIs for a broad range of blood variables for use in clinical health assessments, and (2) compare blood variables for loggerhead turtles residing in coastal foraging grounds and during migration to provide insight into the energetic and physiological status associated with different behavioural states.

## Materials and methods

### Ethics statement

This study is one component of on-going research performed by NOAA Northeast Fisheries Science Center (NOAA NEFSC) and Coonamessett Farm Foundation on loggerhead turtles of the NW Atlantic population. All research was authorized and conducted under the Endangered Species Act (Permits #14 249, #16 556, #18 526).

### Turtle capture and sampling

**Figure 1:**
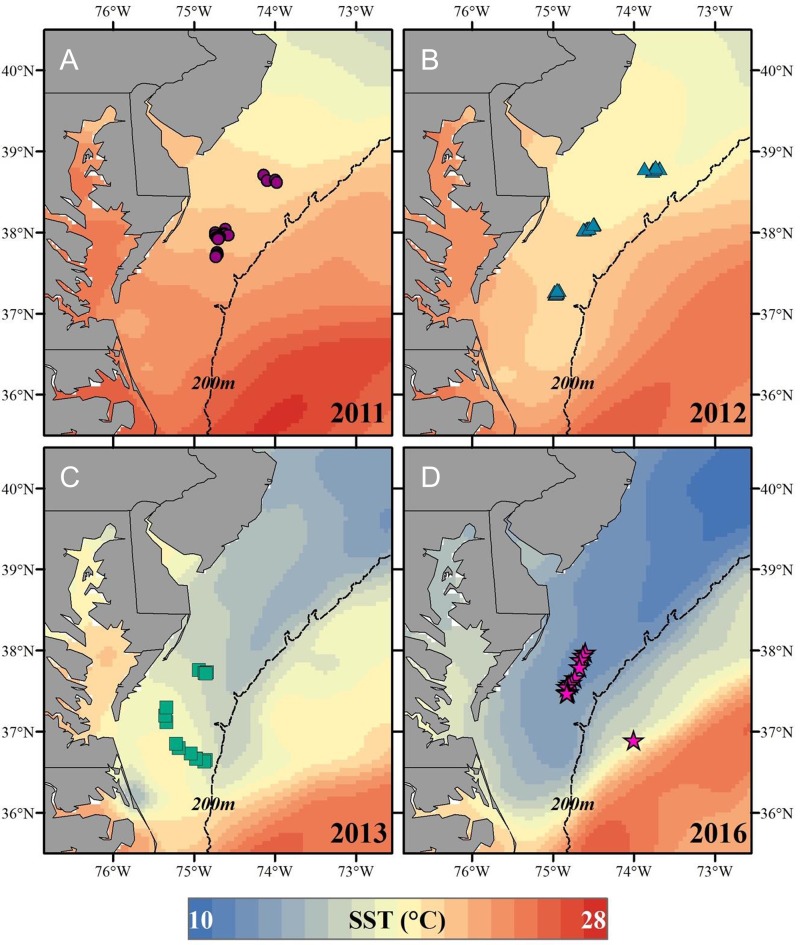
Geographic distribution for all loggerhead turtles (*Caretta caretta*) captured across sampling years (**A-D**: 2011, 2012, 2013, 2016, respectively). The associated average sea surface temperature for each sampling period per year is provided with a colour-coded legend below the map, with each colour representing a single degree C.

Turtles were sampled from May to June in 2011, 2012, 2013 and 2016 along the continental shelf off the Mid-Atlantic coast of the USA (36–39°N, 73–75°W; Figure 1). Individual loggerhead turtles were spotted at-sea while aboard the F/V Kathy Ann, a 91 ft commercial scalloping vessel chartered for this research. To avoid startling the turtle, the research vessel remained situated at a distance, and a small, inflatable boat was deployed with a driver and a netter to capture the turtle. Personnel on-board the research vessel maintained sight of the turtle and directed the small boat to a distance where the netter gained visual contact. The small boat then approached the turtle from behind to avoid startling the animal. When close enough, the netter quickly placed a large dip net in front of the turtle, allowing the turtle to swim forward into the net. After the turtle was netted, it was brought aboard the small boat and transported back to the research vessel. Of the 81 loggerhead turtles sampled, 73 were designated as large juveniles (58.1–80.0 cm SCL, *N* = 66) or sub-adults (80.1–87.0 cm SCL, *N* = 7) according to size classifications previously established (Crouse *et al.*, 1987).

Processing of each turtle involved the collection of a blood sample (see below), core body temperature (*T*) measurement via a soft thermocouple thermistor (Model 8402-00; Cole Parmer Instrument Co., Vernon Hills, IL) inserted 4–8 cm into the cloaca, and SCL_NT (straight carapace length_notch to tip) measurement using calipers. Satellite transmitters (GPS-Argos Satellite Relay Data Loggers; Sea Mammal Research Unit, University of St. Andrews, St Andrews, Fife, KY16 8LB, UK) were attached to the carapace of each turtle as part of a separate study of loggerhead turtle movements and behaviour (Winton *et al.*, 2018); turtles tracked for ≥3 months by satellite telemetry were considered ‘healthy’ and were included in blood biochemistry analysis.

### Blood sample collection and handling

**Figure 2:**
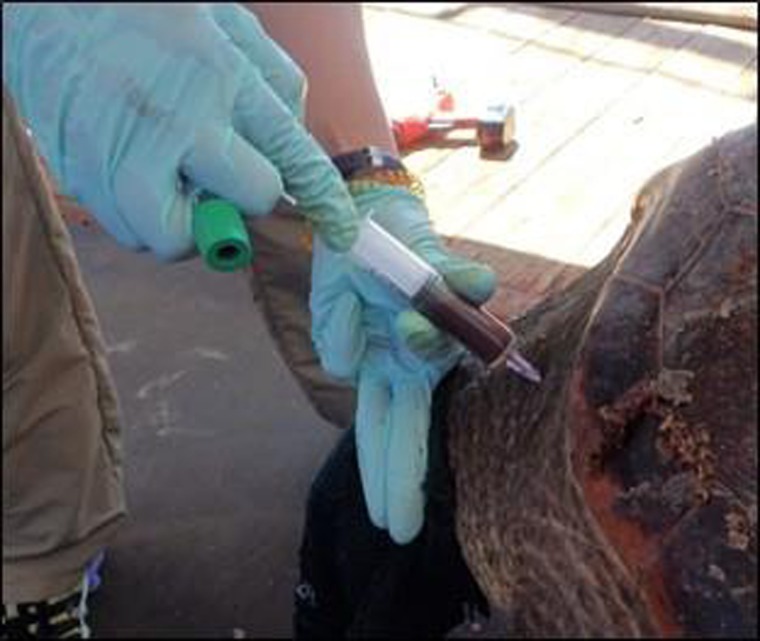
Blood was collected from the dorsal cervical sinus of loggerhead sea turtles using a 1.5″ 20-gauge needle and 12-ml syringe.

Blood samples (12 ml) were obtained from the dorsal cervical sinus using a 1.5′ 20-gauge needle and 12-ml syringe (Figure 2). The sample was immediately divided between green-top tube (GTT) vacutainers containing lithium heparin with no plasma separator. Subsamples were drawn from GTT vacutainers using a 1.5′ 20-gauge needle and 1-ml syringe for analysis via an i-STAT Handheld point-of-care blood analyser (Abbott Point-of Care Inc.; Princeton, NJ). In 2011, 2012 and 2013, additional subsamples were collected for manual determination of packed cell volume (PCV) by centrifugation in haematocrit tubes, determination of total solids by refractometer and preparation for veterinary diagnostic laboratory (VDL) analyses at IDEXX Reference Laboratories (Buzzards Bay, MA). For the latter, plasma (1 ml) was harvested by centrifugation of remaining blood in GTTs and frozen at −18°C, and 1 ml of whole blood in a small GTT was refrigerated. Both the plasma biochemical profile and complete blood count were assessed by VDL analysis within 8 days.

### Biochemistry, blood gas and haematology variables

The i-STAT analyser was used in conjunction with three types of cartridges to measure blood variables. In 2011, CG4+ cartridges (pH, pCO_2_, pO_2_, HCO_3_^−^, TCO_2_, sO_2_, Base Excess, lactate) were loaded with a subsample of whole blood drawn from a GTT vacutainer. In 2012 and 2013, CG8+ cartridges (pH, pCO_2_, pO_2_, HCO_3_^−^, TCO_2_, sO_2_, Base Excess, haematocrit (Hct), haemoglobin (Hgb), sodium (Na), potassium (K), ionized calcium (iCa), glucose (Glu)) were loaded immediately with samples directly taken from turtles and subsamples for CG4+ cartridges were prepared as in 2011. The i-STAT analysis in 2016 used CHEM8+ (TCO_2_, Hct, Hgb, Na, K, chloride (Cl), Anion Gap, iCa, Glu, blood urea nitrogen (BUN), creatinine (Crea)) and CG4+ cartridges, in that order, loaded as subsamples from GTT vacutainers. Previous work has suggested no significant difference between blood variables measured with different types of i-STAT cartridges (Lewbart *et al.*, 2014). Thus, for blood variables with multiple i-STAT measurements, values derived from the first cartridge run were used for assessment of baseline blood biochemistry. The average time lag between blood collection and loading blood into i-STAT cartridges for analysis was 12 min (range 1–137 min).

Blood gas variables (pH, pCO_2_, pO_2_, HCO_3_^−^, iCa, TCO_2_) were assessed at 37°C by the i-STAT instrument, thus, the values were temperature-corrected to each individual turtle’s core body temperature (*T*) based on the published equations listed below.

(1) $${\mathrm{pH}}_{\mathrm{TC}}={\mathrm{pH}}_{37^{{}^{\circ}}C}+0.014\;*\;T$$

(Kraus and Jackson, 1980; Harms *et al.*, 2003)

(2) $${\mathrm{pCO}}_{2\;\mathrm{TC}}={\mathrm{pCO}}_{2\;37^{{}^{\circ}}C}\;*\;10^{\left(-0.019\;*\;T\right)}$$

(Ashwood *et al.*, 1983; Harms *et al.*, 2003)

(3) $${\mathrm{pO}}_{2\;\mathrm{TC}}={\mathrm{pO}}_{2\;37^{{}^{\circ}}C}\;*\;10^{\left(-0.0058\;*\;T\right)}$$

(Ashwood *et al.*, 1983; Harms *et al.*, 2003)

(4) $${\mathrm{HCO}}_{3}^{-}\mathrm{TC}={\alpha \mathrm{CO}}_{2}\;*\;{\mathrm{pCO}}_{2}\;*\;10^{\left(\mathrm{pH}-\mathrm{pKa}\right)}$$

(Stabenau and Heming, 1993; Harms *et al.*, 2003)

where *α*CO_2_ = 9.174 ∗ 10^−2^ – (3.269 ∗ 10^−3^)(T) + (6.364 ∗ 10^−5^)(T^2^) – (5.378 ∗ 10^−7^)(T^3^), and pKa = 6.398 – (1.341 ∗ 10^−2^)(T) + (2.282 ∗ 10^−4^)(T^2^) – (1.516 ∗ 10^−6^)(T^3^) – log_10_ (1.011 + 10^(pH+0.011 ∗ T–10.241)^ + 10^(pH+0.001 ∗ T–8.889)^)

(5) $${\mathrm{iCa}}_{\mathrm{TC}}=\mathrm{iCa}(1+0.53\;\left(\mathrm{pH}-{\mathrm{pH}}_{\mathrm{TC}}\right)$$

(Fogh-Andersen, 1981)

(6) $${\mathrm{TCO}}_{2\mathrm{TC}}={\mathrm{HCO}}_{3\mathrm{TC}}^{-}+({\alpha \mathrm{CO}}_{2}\;*\;{\mathrm{pCO}}_{\mathrm{2TC}})$$

(Abbott Point of Care Inc., 2013).

The plasma biochemical panel used in VDL analysis (IDEXX Reptile Profile #1) provides measurements for the following variables: alkaline phosphatase (ALP), alanine transaminase (ALT), aspartate aminotransferase (AST), creatine kinase (CK), lactate dehydrogenase (LDH), albumin, globulin, A/G ratio, total protein (TP), plasma protein (PP), Glu, cholesterol, total calcium (Ca), phosphorus (P), K, Na and uric acid (UA). The VDL analysis also provides haematology results obtained via laser flow cytometry, optical fluorescence and Laminar Flow Impedance (ProCyte Dx* Haematology Analyzer). The following haematology variables are provided as part of the panel: white blood cell estimate/count (WBC), haematocrit (Hct), % (differential count) heterophils, ABS (absolute count) Heteros, % lymphocytes, ABS Lymphs, % azurophils, ABS Azuros, % eosinophils, ABS Eosinos, % monocytes, ABS Monos and plasma protein (PP). A small number of VDL variables (Na, K, Glu) also were measured by i-STAT. Previous studies have concluded that differences between values derived from VDL analysers and i-STAT are not biologically or clinically significant (Wolf *et al.*, 2008; Atkins *et al.*, 2010). Thus, values obtained in the field using i-STAT were maintained for analysis. This choice minimized the potential for handling or storage effects on blood values. Haematocrit values were derived from i-STAT and VDL, and PCV was determined via manual centrifugation of Hct tubes (Supplementary Table S1). Previously published work has illustrated that Hct values provided by i-STAT are lower than values obtained manually by centrifugation of Hct tubes in loggerhead turtles (Wolf *et al.*, 2008) and hawksbill turtles (Muñoz-Pérez *et al.*, 2017). Based on recommendations by Muñoz-Pérez *et al.* (2017), we include only manually determined Hct values in our analysis.

### Statistical analysis

For a broad assessment of the data, descriptive statistics were calculated for size, core body temperature and blood variables across all four sampling years.

RIs for blood biochemistry, blood gas and haematology variables were estimated using previously published methods (Kelly *et al.*, 2015). Distribution of variables was assessed using histograms and box-plots followed by tests for normality using the D’Agostino-Pearson test. Variables with non-Gaussian distribution were transformed using Box-Cox transformations. Outliers were assessed via Tukey’s outlier test, a more conservative approach than the Dixon–Reed outlier test, based on guidelines provided by the American Society for Veterinary Clinical Pathology (ASVCP), which are in accordance with Clinical Laboratory Standards Institute (CLSI) EP28-A3c guidelines (2.5–97.5 percentiles) (Clinical Laboratory Standards Institute, 2010; Friedrichs *et al.*, 2012). Outliers were removed from PCV (*N* = 1), Lactate (*N* = 1), pH (*N* = 2), pCO_2_ (*N* = 1), pO_2_ (*N* = 1), TCO_2_ (*N* = 1), HCO_3_^−^ (*N* = 1), BEecf (*N* = 2), Na (*N* = 1), Glu (*N* = 1), ALP (*N* = 4), UA (*N* = 2) and PP (*N* = 2) analyses. As recommended by Friedrichs *et al.* (2012), the 95% RIs with associated 90% confidence intervals (CIs) for limits of intervals were estimated via the robust method for variables with 40 ≥ x ≥ 80 samples, and for variables x ≤ 40 the parametric method was used. All RIs were estimated using MedCalc for Windows v17.7.2 (MedCalc Software; Ostend, Belgium).

To assess the time-sensitivity of blood gas measurements (pH, pCO_2_, pO_2_, TCO_2_, HCO_3_^−^), regression analysis was performed on the absolute difference in blood gas values measured by different cartridges (|CG8+—CG4+|) against time elapsed (min) between cartridge loading (*P* ≤ 0.05) for the 2012 dataset.

We used the Mann–Whitney *U* test to compare 21 blood variables for juvenile to sub-adult loggerheads in a nearshore foraging habitat (Kelly *et al.*, 2015) and during migration (our study) to investigate significant differences between behavioural states. *P*-values were adjusted via the Holm–Bonferroni method (*P* ≤ 0.002).

The influence of size (SCL_NT) and core body temperature (*T*) on individual blood biochemistry and haematology variables was assessed by creating a correlation matrix between all measured variables. Then the Spearman rank correlation coefficients and associated *P*-values for correlations between SCL_NT and *T* were extracted from the matrix. Associated *P*-values were adjusted via the Holm–Bonferroni method to reduce the chance of spurious correlations due to Type I error from multiple comparisons (for *T* and SCL_NT, *P* ≤ 0.001).

All statistical analyses excluding calculation of RIs were conducted using Microsoft Excel and R v3.2.0 (The R Foundation for Statistical Computing, Vienna, Austria) through the RStudio interface (R Studio, Boston, MA, USA).

## Results

The median SCL_NT for all turtles combined was 73.7 cm and values ranged from 54.9 to 100.8 cm. Median *T* was 19.6°C with values ranging from 12.3 to 25.3°C. Summary information for each sampling year is presented in Table 1. Basic descriptive statistics (median and range) and RIs for blood biochemistry, blood gas and haematology variables are reported in Table 2.

**Table 1:** Summary of sampling methodology by year (2011, 2012, 2013, 2016), for all loggerhead turtles (*Caretta caretta*) included in data analysis. Sampling methodology is characterized by capture date, key physical attributes and clinical blood analysers used for assessment. SCL_NT and Temperature are presented as Median ± SD. *n = 12

Year	*n*	Capture Dates	SCL_NT (cm)	*T* (cloacal, °C)	*n *CG4+	*n* CG8+	*n* CHEM8+	*n* IDEXX	*n* Hct Tubes
2011	25	June 2–6	72.8 ± 7.3	21.6 ± 1.8	25	0	0	25	24
2012	28	May 31–June 3	76.2 ± 8.6	19.9 ± 0.9	27	28	0	28	28
2013	15	May 21–23	72.9 ± 12.2	17.4 ± 1.9	10	15	0	15	12
2016	13	May 17–20	74.1 ± 10.0*	13.7 ± 1.3	13	0	12	0	0
All years combined	81		73.7 ± 9.2	19.6 ± 3.1	75	43	12	68	64

**Table 2.** Blood analyte values for wild, healthy sub-adult loggerhead turtles captured along the shelf of the Northwest (NW) Atlantic. All blood gas values reported from the present study were measured from venous blood and temperature-corrected by internal cloacal temperature (*T*°C) taken upon landing of individual turtles

Blood variable	Units	*N*^a^	Median (Range)	Lower limit (90% CI)	Upper limit (90% CI)	Source
ABS Azuro	/**μ**l	52	275 (48–1120)	78.4 (59.3–106.0)	729.9 (586.8–889.2)	VDL
ABS Eosino	/**μ**l	56	494 (0–3390)	13.6 (2.4–41.1)	2580.2 (1936.9–3266.2)	VDL
ABS Hetero	/**μ**l	63	3600 (900–8710)	1308.1 (1064.3–1588.5)	8400.9 (7246.2–9586.6)	VDL
ABS Lymph	/**μ**l	64	5770 (240–10 800)	429.8 (0–1416.5)	11 034.3 (10 121.9–11 841.2)	VDL
ABS Mono	/**μ**l	22	630 (0–2664)	7.2 (0–67.1)	2796.6 (1865.6–3974.6)	VDL
AG Ratio^b^		61	0.4 (0.2–0.5)			VDL
ALP	U/l	61	14 (5–84)	3.5 (2.1–5.2)	22.9 (20.8–24.7)	VDL
ALT^b^	U/l	61	1 (0–36)			VDL
Albumin^b^	g/dl	61	1.0 (0.5–1.9)			VDL
Anion Gap	mmol/l	12	10 (−3–16)			iS–C^3^
AST	U/l	61	118 (71–1213)	75.9 (71.8–80.9)	432.7 (264.7–2674.8)	VDL
Azurophils^b^	%	52	2 (1–8)			VDL
BEecf	mmol/l	79	8 (−11–23)	−5.3 (−7.4–3.1)	21.2 (18.9–23.2)	iS–C^1,2^
BUN	mmol/l	12	9.0 (3.1–16.2)			iS–C^3^
Ca	mg/dl	61	7.4 (5.4–12.0)	5.3 (5.0–5.7)	10.3 (9.6–10.9)	VDL
Cholesterol	mg/dl	61	104 (42–187)	34.7 (23.6–47.2)	167.9 (155.0–180.5)	VDL
CK	U/l	61	928 (285–2759)	323.3 (267.9–394.1)	2375.8 (2013.5–2810.2)	VDL
Cl	mmol/l	12	105 (96–113)			iS–C^3^
Creatinine	μmol/l	12	20 (18–27)			iS–C^3^
Eosinophils	%	56	4 (0–16)	0 (0–0)	13.7 (11.3–15.5)	VDL
Globulin	g/dl	61	2.9 (1.7–4.6)	1.7 (1.5–2.0)	4.0 (3.8–4.3)	VDL
Glucose	mg/dl	55	74 (47–332)	39.3 (33.6–46.2)	109.0 (100.9–116.5)	iS–C^2,3^
Heterophils	%	64	33 (14–95)	14.8 (13.1–16.9)	91.6 (74.8–110.5)	VDL
HCO_3_^−^	mmol/l	65	37.9 (21.2–54.7)	23.2 (20.6–26.0)	52.6 (50.1–54.9)	iS–C^1,2^
iCa	mmol/l	54	0.78 (0.55–1.32)	0.59 (0.57–0.62)	1.23 (1.08–1.41)	iS–C^2,3^
K	mmol/l	55	3.4 (2.6–4.8)	2.5 (2.3–2.7)	4.4 (4.2–4.6)	iS–C^2,3^
Lactate	mmol/l	75	5.82 (0.30–19.06)	0 (0–0.47)	13.07 (11.70–14.19)	iS–C^1^
LDH	IU/l	61	58 (1–474)	2.6 (1.2–5.8)	323.0 (241.0–418.5)	VDL
Lymphocytes	%	64	54 (4–78)	17.2 (9.9–25.1)	90.0 (84.1–96.3)	VDL
Monocytes	%	24	6 (0–18)	0 (0–0)	16.1 (13.3–19.0)	VDL
Na	mmol/l	55	147 (136–163)	139.2 (137.3–141.1)	155.3 (153.7–156.9)	iS–C^2,3^
P	mg/dl	61	5.4 (2.9–10.6)	3.0 (2.7–3.4)	9.3 (8.4–10.2)	VDL
pCO_2_^c^	mmHg	65	36.1 (21.5–55.9)	20.7 (18.9–23.0)	58.9 (54.8–62.7)	iS–C^1,2^
PCV/Hct	%	54	37 (28–68)	27.0 (25.4–28.6)	46.4 (44.6–48.1)	T
pH		65	7.521 (7.315–7.675)	7.330 (7.295–7.367)	7.701 (7.670–7.727)	iS–C^1,2^
Plasma protein	g/dl	63	4.2 (2.5–33.2)	2.4 (2.1–2.7)	5.9 (5.6–6.3)	VDL
pO_2_	mmHg	65	67 (39–103)	39 (35–44)	94 (89–98)	iS–C^1,2^
TCO_2_	mmol/l	65	40 (22–56)	24 (22–27)	55 (53–58)	iS–C^1,2^
Total protein	g/dl	61	3.9 (2.4–5.9)	2.4 (2.2–2.7)	5.5 (5.2–5.8)	VDL
Total solids		63	5.0 (3.0–7.4)	2.8 (2.2–3.1)	7.1 (6.6–7.5)	R
Uric acid	mg/dl	61	1.3 (0–3.3)	0.1 (0–0.3)	2.4 (2.2–2.6)	VDL
WBC(count)	THOUS	64	11.7 (3.5–15.0)	4.8 (3.8–6.2)	17.3 (16.2–18.2)	VDL
WBC(max)	THOUS	64	12.7 (4.5–16.0)	5.8 (4.8–7.3)	18.4 (17.2–19.3)	VDL
WBC(min)	THOUS	64	10.7 (2.5–14.0)	3.8 (2.8–5.2)	16.3 (15.2–17.2)	VDL

‘Source’ is iS = i-STAT followed by cartridge type (CG4+ = C^1^, CG8+ = C^2^, CHEM8+ = C^3^); VDL = veterinary diagnostic laboratory; T = Hct tubes; R = refractometer.

^a^Observations before outlier removal.

^b^Reference intervals and confidence intervals could not be calculated by robust methods.

^c^Non-Gaussian distribution following Box-Cox transformation.

When the difference in blood gas values obtained with different cartridges (|CG8+—CG4+|) were plotted against time elapsed between cartridge loading, weak but statistically significant relationships were found for pCO_2_ (*P* = 0.013, *R*^2^ = 0.2134), TCO_2_ (*P* = 0.011, *R*^2^ = 0.2233), and HCO_3_^−^ (*P* = 0.024, *R*^2^ = 0.1810). The other blood gas variables did not demonstrate statistically significant relationships with time elapsed between loading cartridges. These statistically significant results provide support for utilizing the first cartridge run for analysis.

The results from our study are presented alongside values for loggerhead turtles in nearshore foraging grounds (Kelly *et al.*, 2015) to facilitate comparisons between different geographic locations (Table 3). Comparisons with additional studies are presented in Supplementary Table S2. The results of the Mann–Whitney *U* test indicated that 14 out of 21 blood variables were significantly different (*P* ≤ 0.002) between turtles resident in nearshore foraging habitats (SCL range 50.4–80.6 cm, Kelly *et al.*, 2015) and migratory turtles in our study (SCL range 54.9–100.8 cm). Median values for PCV/Hct, TP, globulin, ABS Azuros, ABS Lymphs and UA were all higher in migrators while median values for Glu, Na, K, P, Cl, AST, ABS Monos and BUN were all lower in migrators compared with resident turtles.

**Table 3:** Comparison of blood variables between migratory and resident turtles This table shows the median and range for each blood variable for this study (migratory turtles) and Kelly *et al.* (2015, resident turtles) and the Holm–Bonferroni adjusted *P*-values obtained from the Mann–Whitney *U* test when comparing results. Significance was set at *P* ≤ 0.002. *Signifies blood variables that differ significantly between migratory and residential turtles. ^a^Signifies a value from Kelly *et al.* (2015) that was converted to the units used in our study. Please see Table 2 for sample sizes for each variable for migratory turtles. For resident turtles *N* = 191, unless denoted^b^ which signifies *N* = 190

	Migratory median (Range)	Resident median (Range)	*P*-values adj
SCL_NT (cm)	73.7 (54.9–100.8)	63.3 (50.4–85.6)	1.59E–14*
ABS Azuros (/μl)	275 (48–1120)	0 (0–1200)^b^	4.84E–15*
ABS AzurosMonos (/μl)	300 (0–2960)	210 (0–1650)^b^	2.36E–02
ABS Eosinos (/μl)	494 (0–3390)	300 (0–4800) ^b^	2.02E–02
ABS Hets (/μl)	3600 (900–8710)	4700 (0–21 600)^b^	7.44E–02
ABS Lymph (/μl)	5770 (240–10 800)	3400 (600–9200)^b^	3.61E–07*
ABS Monos (/μl)	630 (0–2664)	1400 (0–1600)^b^	4.31E–05*
Albumin (g/dl)	1.0 (0.5–1.9)	1.1 (0.4–1.7)	1.00E+00
AST (U/l)	118 (71–1213)	161.0 (50.0–390.0)	1.43E–05*
BUN (mmol/l)	9.0 (3.1–16.2)	23.6 (6.1–67.8)	3.61E–06*
Ca (mg/dl)	7.4 (5.4–12.0)	7.6 (5.2–11.6)	1.00E+00
CK (U/l)	928 (285–2759)	1034.0 (153.0–13 310.0)	1.00E+00
Cl (mmol/l)	105 (96–113)	115.0 (101.0–129.0)	5.52E–05*
Globulin (g/dl)	2.9 (1.7–4.6)	2.4 (1.3–4.6)^a^	1.65E–07*
Glucose (mg/dl)	74 (47–332)	104 (45.233)	8.71E–13*
K (mg/dl)	3.4 (2.6–4.8)	4.2 (2.5–6.1)	4.91E–12*
Na (mEq/l)	147 (136–163)	156.0 (145.0–168.0)	4.84E–15*
P (mg/dl)	5.4 (2.9–10.6)	6.8 (3.7–11.1)^a^	4.00E–09*
PCV (%)	37 (28–68)	31.0 (9.0–40.0)^b^	6.86E–15*
Total protein (g/dl)	3.9 (2.4–5.9)	3.5 (2.1–6.0)^a^	5.37E–04*
UA (mg/dl)	1.3 (0–3.3)	0.8 (0.1–2.8)^a^	1.53E–04*
WBC (THOUS.)	11.7 (3.5–15.0)	9.0 (2.0–27.0)^b^	1.59E–01

Spearman rank correlation coefficients illustrated that Na (*ρ* = 0.54, *P* ≤ 0.001), pCO2 (*ρ *= −0.52, *P* ≤ 0.001), Lactate (*ρ* = 0.45, *P* ≤ 0.001) and LDH (*ρ* = 0.50, *P* ≤ 0.001) were significantly correlated with *T*. None of the blood variables measured were significantly correlated with SCL_NT.

## Discussion

The migratory sample population in our study and resident loggerhead turtles sampled at seasonal (May–November) neritic foraging habitats in Core Sound, North Carolina (Kelly *et al.*, 2015) are of similar size (juveniles to sub-adults) and likely both derived from the NWA DPS (Winton *et al.*, 2018), thus permitting a comparison of RIs at the intra-population level during different behavioural and physiological states. Of the 21 blood variables included in our comparison, 14 variables showed statistically significant differences. Additionally, we found that *T* was significantly correlated with blood variables related to metabolic status. This helps validate the practice of considering ecological and biological processes when establishing RI values for a species.

Given the time of year in which sampling occurred, juvenile and sub-adult loggerhead turtles sampled in our study likely were migrating from overwintering grounds in North Carolina or further south to seasonal foraging grounds at higher latitudes (Winton *et al.*, 2018). These younger age classes are not undertaking migration for breeding and reproductive purposes, as the adults do, rather they are driven to migrate due to seasonality and spatiotemporal distribution of resources (Chambault *et al.*, 2015). Previous studies have illustrated that marine turtles may show preference for specific foraging grounds over others and exhibit strong site fidelity to those foraging areas, with spatial ranges found to be as small as < 5 km^2^ in some loggerhead populations (Thomson *et al.*, 2012; Carman *et al.*, 2016; Winton *et al.*, 2018). Ceriani *et al.* (2014) identified that inter-individual isotopic variance in loggerhead turtles may be reflective of differences in behavioural preference for specific migratory and foraging grounds rather than dietary trophic level or individual physiological variation as previously assumed (Vander Zanden *et al.*, 2010). The loggerheads captured for our study were utilizing a major migratory corridor that has been documented in earlier studies for both juveniles and adults (Winton *et al.*, 2018), but the physiological status of turtles along this migratory route had not been described previously.

Understanding the migratory physiology of marine turtles, specifically juveniles and sub-adults, is a difficult endeavour given the logistic difficulties of locating and sampling healthy individuals, as well as the limited capacity for continued monitoring of turtles following initial capture and sampling. Our study provides the first documentation of blood chemistry and haematology for loggerhead turtles during northward spring (May–June) migrations in the NW Atlantic and, therefore, provides a unique opportunity to investigate the physiological status of this species in a temperate latitude offshore habitat. Furthermore, our data permit an assessment of the physiological differences between migratory and resident juvenile loggerhead turtles. Comparisons between these different behavioural states can provide insight regarding energetic status and whether or not juvenile turtles rely on endogenous energy stores for the migratory trip (Åkesson and Hedenström, 2007), as do other long-distance migrators. Additionally, information about metabolic demands and strategies, and how metabolism may be impacted by variable temperatures experienced over the course of migration, may be gained through explorations of blood biochemistry. Finally, assessments of health status may be facilitated by haematology data.

### Comparison of migrating vs. resident turtles

Migratory turtles had significantly lower Glu, blood ions (Na, K, P and Cl) and BUN. Stamper *et al.* (2005) also noted a decrease in Glu, blood ions (Na, K, Ca and Cl) and BUN in loggerhead turtles migrating through Pamlico and Core Sound, NC in late fall compared with summer resident turtles at these sites, and hypothesized that the differences in these blood variables reflected a less active foraging pattern and decreased waste production in migrators. Adult female loggerheads are aphagic and rely on endogenous energy stores during their extensive breeding migrations (Bonnet *et al.*, 1998), but much less is known regarding the foraging patterns of juvenile and sub-adult loggerheads during migration. Snover *et al.* (2010) noted that the diet of loggerhead turtles in neritic habitats is more nutrient dense than that in oceanic habitats. Foraging opportunities may be limited along offshore migratory routes, or juvenile to sub-adult turtles may prioritize travelling over foraging during directed long-distance movements.

Interestingly, we found that UA was significantly higher in migrators compared with turtles in nearshore foraging habitats. Glomerular filtration rate (GFR) of UA remains constant for birds during long-distance migrations (Landys *et al.*, 2005; Gerson and Guglielmo, 2013); if the same is true of migratory marine turtles, then increased production of UA due to an increase in protein catabolism, linked with unchanging UA clearance rates, would result in higher levels of plasma UA (Bairlein *et al.*, 2015). Reliance on protein catabolism may increase during long-distance migrations as carbohydrate and lipid energy stores are depleted with high and continuous levels of energy expenditure (Martin *et al.*, 2015). Furthermore, water produced from protein catabolism may help offset respiratory water loss during periods of sustained activity (Gerson and Guglielmo, 2011). The relative importance of different endogenous fuel stores in migrating turtles is a topic worthy of further investigation (Jenni and Jenni-Eiermann, 1998; Guglielmo, 2010; Bairlein *et al.*, 2015). Although UA has traditionally been thought of as a metabolic end waste product that is not biologically useful (Keilin, 2008), more recent research has demonstrated beneficial antioxidant and neuroprotective effects from circulatory UA (Johnson *et al.*, 2009; Álvarez-Lario and Macarrón-Vicente, 2010). These features of UA might be biologically significant for migratory animals should they incur oxidative and metabolic stress from extensive fuel usage and depletion (Skrip *et al.*, 2015).

We also documented lower levels of AST in migrators compared with resident turtles. Lower levels of AST are associated with uremia, the pathological condition of excessive nitrogenous waste in the blood (Warnock *et al.*, 1974), and are generally correlated with higher levels of UA, BUN and P in human patients (Gao *et al.*, 2000). In contrast, we observed significantly lower levels of BUN and P in migrators compared with resident turtles in our study. Migratory animals may have adaptations to regulate nitrogen metabolism during conditions of decreased food intake to allow for more efficient recycling of BUN for amino acid/protein synthesis, as has been documented for fasting, hibernating mammals (Stenvinkel *et al.*, 2013). If marine turtles are capable of employing such mechanisms, this could explain the discrepancy in trends for UA and BUN levels between migratory and resident turtles.

The median for PCV/Hct for migratory turtles in the Mid-Atlantic was significantly higher than that of resident turtles in Core Sound; however, migratory turtles also had a greater median SCL_NT, which could affect interpretation of the observed difference. As documented in previous studies (Frair, 1977; Frair and Shah, 1982; Bolten and Bjorndal, 1992; Osborne *et al.*, 2010; Stacy *et al.*, 2018), there is a positive correlation between body size and blood cell characteristics, including size and quantity of cells. That said, an increase in PCV/Hct also could provide migratory loggerhead turtles with enhanced capacity for oxygen delivery to support sustained, aerobic activity during long-distance migration (Krause *et al.*, 2016).

Total protein and globulin were higher in migratory loggerheads compared with nearshore residents. Markedly increased levels of TP and globulin have been documented in nesting marine turtles and are hypothesized to be indices of vitellogenesis and folliculogenesis (Casal *et al.*, 2009), but this cannot explain the trends observed in juvenile and sub-adult turtles. Hyperproteinemia can occur in response to dehydration (Manning, 1998a, b), and this interpretation is supported by the higher PCV/Hct observed for migrating turtles; however, dehydration does not occur in avian long-distance migrators. The ability of birds to maintain water balance during migration, despite high levels of respiratory water loss associated with elevated metabolic rates, is due to increased water produced from protein catabolism (Gerson and Guglielmo, 2011). The significantly higher UA levels in migrating turtles suggests that protein catabolism may be occurring, but perhaps the resultant water production is not sufficient to offset sources of water loss during migration. Gicking *et al.* (2004) found that values for beta-globulin in Atlantic loggerheads were significantly higher in adult turtles compared with juveniles, so higher levels of globulin in migrators may simply reflect the larger size of migrators compared with resident turtles; however, Gicking *et al.* (2004) also found significant differences between sexes, thus, determining the physiological basis for beta-globulin variance amongst age and sex classes requires further research.

Migratory turtles had higher levels of ABS Azuros and ABS Lymphs, and lower levels of ABS Monos compared with turtles resident at nearshore foraging grounds. Stacy *et al.* (2011) recommends combining ABS Azuros and ABS Monos in all reptile taxa excluding snakes, as these leukocytes are morphologically, and likely functionally, similar in most reptile species; upon combining these two leukocytes the difference between migratory versus resident turtles is not statistically significant. Nevertheless, the difference in ABS Lymphs remains. Elevated levels of lymphocytes typically indicate inflammation or infection in reptiles (Stacy *et al.*, 2011), but it is unclear why migratory turtles would be more prone to infection. Some work has demonstrated that migration may increase the risk of spreading infectious diseases due to anthropogenically created migratory stopover hotspots generated by habitat loss; however, other studies indicate that migration might offer an evolutionary benefit against accumulation of parasites due to spatiotemporal avoidance of areas with high infection potential, culling of infected individual through the process of migration, or recovery from infection during the process of migration (Shaw and Binning, 2016). An alternate way of looking at this result is that lower levels of lymphocytes documented in resident turtles compared with migrators may indicate that residents are experiencing immunosuppression due to increased glucocorticoid circulation in response to in-shore stressors (Aguirre *et al.*, 1995; Milton and Lutz, 2003; Tarlow and Blumstein, 2007). In this case, the lymphocyte profile exhibited by migratory turtles from our study would be the non-pathologic immunological state.

### Temperature and size effects

We found that *T* was significantly correlated with blood variables related to metabolic status. Both venous and arterial blood can reflect aspects of metabolic status, including metabolic acidosis (Brandenburg and Dire, 1998); we used venous blood in our study. Lactate was positively correlated with *T* (12.3–25.3°C) in migrating turtles; this is in contrast with previous findings for captive sub-adult loggerhead turtles, in which plasma lactate values were independent of *T* (15–30°C) until especially low temperatures were achieved (10°C), at which point an elevation in lactate occurred (Lutz *et al.*, 1989). The positive correlation observed in our study may be due to stable lactate clearance times (Gerson and Guglielmo, 2013) combined with differences in metabolic demand and capacity at different temperatures for ectothermic turtles; increased anaerobic capacity at warmer temperatures could result in higher levels of circulating lactate, particularly in response to vigorous activity (Martin *et al.*, 2015).

Concurrent with the increase in lactate with *T*, we also observed a significant positive correlation between LDH and *T*. Enzymes, such as LDH, that catalyse intracellular biochemical reactions are released into the bloodstream due to cell turnover. If higher levels of enzyme are present in the cells, as may be expected with increased metabolic capacity at higher *T*, this will also be reflected by plasma levels of the enzyme. Metabolic pathways utilized for lactate clearance by migratory or endurance-exercised animals include the resynthesis of glycogen stores (gluconeogenesis) and direct lactate oxidation (Martin *et al.*, 2015); both pathways utilize the LDH enzyme to convert lactate to pyruvate, which serves as substrate for subsequent biochemical reactions. If migratory turtles decrease food consumption, there may be a preference towards gluconeogenesis as a means to replenish glucose and glycogen stores, given the importance of these substrates for maintaining the vital functions of certain organs (Tavoni *et al.*, 2013). Many ectothermic animals (most herpetofauna and fishes) store lactate intramuscularly for the synthesis of glycogen (Gleeson, 1996), and previous work with lizards has demonstrated that the primary fate of lactate produced during exercise is gluconeogenesis rather than direct oxidation (Gleeson and Dalessio, 1989).

We found a significant negative correlation pCO_2_ with *T*. This is in contrast to the results of Lutz *et al.* (1989), which reported a positive correlation between pCO_2_ and *T* in loggerhead plasma. Lutz *et al.* (1989) interpreted this positive correlation as a reflection of maintenance of constant relative alkalinity of the blood at different temperatures. The discrepancy between previous laboratory studies and our results could be due to differences in metabolic demand and acid-base maintenance for migratory turtles. If anaerobic capacity increases with increasing *T*, as suggested by our LDH results and locomotory performance studies (Elnitsky and Claussen, 2006), this may result in higher circulating levels of lactate and potential disturbances to blood pH during periods of activity. Harms *et al.* (2003) found that metabolic acidosis associated with lactate accumulation due to capture stress could be mitigated via hyperventilation and a concurrent decrease in blood pCO_2_ in loggerhead turtles. A similar phenomenon may occur with shifts between aerobic and anaerobic metabolic pathways as a result of variable activity intensity at higher temperatures.

The positive correlation between plasma Na and *T* has been documented previously in loggerhead turtles (Lutz and Dunbar-Cooper, 1987). Hypernatremia may occur in association with dehydration (Morley, 2015), and the potential for dehydration in migrating turtles may increase with *T* as metabolic and respiratory rates increase.

### Conclusions and conservation implications

As indicated by the number of significantly different blood variables between migratory and residential loggerhead turtles, the relevance of assessing this population during all its behavioural states is of great importance, particularly if blood variables are to be used for assessing physiological impacts of anthropogenic disturbances. As a case study, we can consider the physiological impacts for sea turtles that interact with fisheries. Loggerhead turtles that are a part of the NWA DPS are susceptible to pressures from the commercial gillnet fisheries (Murray and Orphanides, 2013) and the dredge and bottom trawl fisheries for scallops and fishes conducted in the Mid-Atlantic (Murray, 2011; Warden, 2011). Sea turtles entangled in fishing gear may struggle to reach the surface to breathe, and experience respiratory and metabolic disturbances due to prolonged submergence. Signs of respiratory and metabolic distress could be revealed by assessing blood gases, pH, bicarbonate and lactate (Williard *et al.*, 2015). Furthermore, blood cell counts and enzyme profiles may provide insight into injuries sustained by the animal while entangled. Establishment of baseline RIs for migratory loggerheads in the Mid-Atlantic will facilitate future studies of the impacts of anthropogenic threats, such as fisheries interactions, on health status and post-release survival of loggerhead turtles in this region.

Fisheries interactions are just one of many anthropogenic factors that may impact migratory marine vertebrates (Lennox *et al.*, 2016). Climate change is of great concern, due to the potential effects on physiological and ecological aspects of migration. Shifts in thermal regimes have the potential to influence the energetic costs of migration, especially for poikilothermic animals like sea turtles. Direct effects of temperature on metabolic physiology of marine turtles (Davenport, 1997) have the potential to influence diving behaviour, which is often limited by thermo- and haloclines (Arendt *et al.*, 2012; Chambault *et al.*, 2015). Turtles may also be affected by projected changes in ocean currents, as cost-effective usage of passive transport may be important for documented resting behaviours exhibited by migrating loggerhead turtles at night (Dujon *et al.*, 2014). Oceanographic changes may cause shifts in food type and availability which can influence rates of growth and development (Hawkes *et al.*, 2009), and trophic mismatch between energy requirements and availability of suitable resources may become a factor that influences survivorship (Edwards and Richardson, 2004). Migrating sea turtles may lose ephemeral foraging patches, necessitating a change in behaviour to suit changing climate conditions.

Identification of unique aspects of the biochemical and haematological profiles for sea turtles at the intra-population level allows more detailed and in-depth conservation efforts to be implemented through contextualization of the physiology of different behavioural states. By using RIs to provide a physiological basis for the behavioural state of migratory loggerhead turtles at present, clinicians and managers alike can make more confident conservation decisions in the future based on preserving the physiological migratory phenotypes that are currently expressed.

## Supplementary Material

Supplementary Data
