# Supplementary material for: Blood biochemistry and haematology of migrating loggerhead turtles (Caretta caretta) in the Northwest Atlantic: reference intervals and intra-population comparisons
Source: Conserv Physiol. 2019 Feb 7;7(1):coy079. doi: 10.1093/conphys/coy079 (PMC6366141; doi:10.1093/conphys/coy079)
Supplement: Supplementary Data [file coy079_yang_supplementarydata_6dec18.docx]

**Supplementary Data**

Table 1. Comparison of PCV/Hct (%) values (written as Mean ± SD (Range)) derived from three different blood analysis techniques.

| **Methodology** | **PCV/Hct (%)** |
| --- | --- |
| Hematocrit Tubes | 36 ± 5 (28-46) |
| i-STAT CG8+ cartridge | 31 ± 4 (23-40) |
| IDEXX Veterinary Diagnostic Blood Profile | 39 ± 5 (30-50) |

Table 2. Blood analyte values for wild, healthy sub-adult loggerhead turtles captured on the shelf of the Northwest (NW) Atlantic. All blood gas values reported from the present study were measured from venous blood and temperature-corrected by internal cloacal temperature (°C) taken upon landing of individual turtles. Values from the present study are presented alongside literature values for wild, healthy loggerhead turtles of mixed age classes in the NW Atlantic, the Northeast Atlantic, and the Mediterranean Sea. Literature values for blood gas and biochemical variables in venous blood from captive, healthy loggerhead turtles are reported (Lutcavage *et al.* 1987).

| **Reference** | **This Study** | **Kelly *et al.* 2015** | **Deem *et al*. 2009** | **Stacy *et al*. 2018[a]** | **Stacy  *et al*. 2018 [b]** | **Delgado *et al*. 2011** | | **Gelli *et al*. 2009** | **Basile *et al*. 2012[a]** | **Basile *et al*. 2012[b]** | **Lutcavage *et al.*  1987** |
| --- | --- | --- | --- | --- | --- | --- | --- | --- | --- | --- | --- |
| Location | MAB; neritic bordering oceanic | NC, USA; neritic | FL/GA, USA; neritic | FL, USA; neritic | Azores; oceanic | Madeira; oceanic | | Pelagie Islands, Italy Mediterranean Sea; oceanic | Mediterranean Sea; unknown | Mediterranean Sea; unknown | Captive-bred |
| Date | May-Jun 11-16 | May-Nov 04-07 | May-Sep 00-04 | Mar 92-Feb 93 | Nov 90 | May-Jul 06 | | Sep 05 | 02-09 | 02-09 | NA |
| SCL (cm) | 73.7 (54.9-100.8) | 64 (50-81) | 65 ± 7 (52-88)^a^ | 82 (46-108) | 39 (18-60) | 37 (20-52) | | NA | 16-41 | 52-56 | NA |
| Life Stage^b^ | 5 J, 74 LJ/SA, 1 A, 1 U | 191 J/LJ | 35 SA, 5 A | J, A | 28 J | 27 J | | SA | 17 J | 7 A | 8 J |
| *N* | 12-81 | 190-191 | 12-39 | 165-168 | 28 | 4-27 | | 65 | 17 | 7 | 8 |
| **Blood**  **Variable** |  |  | **Mean ± SD (Range)** |  |  |  | |  |  |  |  |
|  | **Median (Range)** | **Median (Range)** | **OR Median (10-90% quartiles)** | **Median (Range)** | **Median (Range)** | **Median (Range)** | | **Mean ± SD (Range)^f^** | **Mean ± SD (Range)** | **Mean ± SD (Range)** | **Mean** |
| ABS Azuro (/**µ**L) | 275 (48-1120) | 0 (0-1200) | NA | NA | NA | NA | | NA | NA | NA | NA |
| ABS Eosino (/**µ**L) | 494 (0-3390) | 300 (0-4800) | NA | NA | NA | NA | | NA | NA | NA | NA |
| ABS Hetero (/**µ**L) | 3600 (900-8710) | 4700 (0-21600) | NA | NA | NA | NA | | NA | NA | NA | NA |
| ABS Lymph (/**µ**L) | 5770 (240-10800) | 3400 (600-9200) | NA | NA | NA | NA | | NA | NA | NA | NA |
| ABS Mono (/**µ**L) | 630 (0-2664) | 1400 (0-1600) | NA | NA | NA | NA | | NA | NA | NA | NA |
| A:G Ratio^b^ | 0.4 (0.2-0.5) | NA | NA | 0.29 (0.11-0.62) | 0.42 (0.3-0.5) | NA | | 0.35 ± 0.08 (0.23-0.53) | NA | NA | NA |
| ALP (U/L) | 14 (5-84) | NA | NA | 13 (3-76) | 26 (11-52) | 68 (51-120) | | 59.53 ± 31.90 (17-129) | NA | NA | NA |
| ALT^b^ (U/L) | 1 (0-36) | NA | 16 ± 6 (0-29) | 1 (0-12) | 1 (0-28) | NA | | 13.32 ± 25.53 (1-104) | NA | NA | NA |
| Albumin^b^ (g/dl) | 1.0 (0.5-1.9) | 1.1 (0.4-1.7) | 1.3 ± 0.3 (0.8-1.6) | 0.9 (0.3-1.8) | 1.1 (0.7-1.3) | 1.3 (1.0-2.0) | | 1.10 ± 0.25 (0.57-1.60)^e^ | NA | NA | NA |
| Anion Gap (mmol/L) | 10 (-3-16) | NA | NA | NA | NA | NA | | NA | NA | NA | NA |
| AST (U/L) | 118 (71-1213) | 161 (50-390) | 165 (2-255) | 186 (39-951) | 148 (94-287) | 79 (13-238) | | 468.00 ± 557.96 (120-3478) | NA | NA | NA |
| Azurophils^b^ (%) | 2 (1-8) | NA | NA | NA | NA | NA | | NA | NA | NA | NA |
| BEecf (mmol/L) | 8 (-11-23) | NA | NA | NA | NA | NA | | NA | NA | NA | NA |
| BUN (mg/dl)^c^ | 25.2 (8.7-45.4)^c^ | 94.4 (33.0-175.9) | 82.9 (1.1-107.0) | 35.5 (2-125) | 77.5 (44-82) | 201.2 (62.1-344.5) | | NA | NA | NA | NA |
| Ca (mg/dl) | 7.4 (5.4-12.0) | 7.6 (5.2-11.6) | 7.6 (5.6-8.4) | 6.9 (2.2-17.1) | 7.3 (4.7-10.9) | 5.1 (3.1-7.1) | | 6.32 ± 1.04 (4.32-10.32)^f^ | NA | NA | NA |
| Cholesterol (mg/dl) | 104 (42-187) | NA | 75.0 (45.2-200.3) | 155 (25-494) | 163.5 (92-361) | 101 (60-200) | | 76.83 ± 32.05 (30.89-166.02)^f^ | NA | NA | NA |
| CK (U/L) | 928 (285-2759) | 1034 (153-13310) | 534 (3-1899) | NA | NA | NA | | 3703.89 ± 3003.35 (0-8620) | NA | NA | NA |
| Cl (mmol/L) | 105 (96-113) | 115 (101-129) | 130 ± 11 (107-158) | 118 (108-127) | 113 (103-123) | 116 (100-136) | | NA | NA | NA | NA |
| Creatinine (mg/dl)^d^ | 0.3 (0.2-0.3)^d^ | NA | 0.3 (0.1-0.5) | 0.3 (0.1-0.7) | 0.2 (0.1-0.3) | NA | | 0.05 ± 0.46 (0.01-0.10)^d^ | NA | NA | NA |
| Eosinophils (%) | 4 (0-16) | NA | NA | NA | NA | NA | | NA | 2.6 ± 2.3 (1.0-6.0) | 2.3 ± 1.9 (1.0-4.5) | NA |
| Globulin (g/dl) | 2.9 (1.7-4.6) | 2.4 (1.3-4.6) | 2.9 ± 0.9 (1.0-4.0) | 3.2 (1.3-5.9) | 2.6 (1.6-3.4) | NA | | 3.32 ± 1.06 (1.76-6.45)^g^ | NA | NA | NA |
| Glucose (mg/dl) | 74 (47-332) | 104.4 (45.0-232.2) | 106.2 ± 19.8 (70.2-136.8) | 95 (54-171) | 114 (88-174) | 132 (71-197) | | 109.19 ± 50.81 (37.84-215.86)^f^ | NA | NA | NA |
| Heterophils (%) | 33 (14-95) | NA | NA | NA | NA | NA | | NA | 62.4 ± 13.0 (41.0-75.0) | 52.6 ± 25.7 (9.9-78.0) | NA |
| HCO_3_^-^ (mmol/L) | 36.7 (20.0-50.9) | NA | NA | NA | NA | NA | | NA | NA | NA | NA |
| iCa (mmol/L) | 0.78 (0.55-1.32) | NA | NA | NA | NA | NA | | NA | NA | NA | NA |
| K (mmol/L) | 3.4 (2.6-4.8) | 4.2 (2.5-6.1) | 5.1 ± 2.0 (3.3-13.9) | 4.2 (2.2-6.5) | 3.7 (3.2-4.1) | 4.5 (3.7-7.3) | | NA | NA | NA | NA |
| Lactate (mmol/L) | 5.82 (0.30-19.06) | NA | NA | NA | NA | NA | | NA | NA | NA | NA |
| LDH (IU/L) | 58 (1-474) | NA | 572 (6-1376) | 92 (24-403) | 61 (19-139) | NA | | 461.32 ± 408.50 (22-1876) | NA | NA | NA |
| Lymphocytes (%) | 54 (4-78) | NA | NA | NA | NA | NA | | NA | 32.4 ± 12.9 (23.0-55.0) | 42.3 ± 25.3 (20.0-85.4) | NA |
| Monocytes (%) | 6 (0-18) | NA | NA | NA | NA | NA | | NA | 2.6 ± 2.1 (1.0-6.0) | 1.8 ± 1.9 (0.2-5.0) | NA |
| Na (mmol/L) | 147 (136-163) | 156 (145-150) | 156 ± 11 (135-175) | 158 (149-179) | 155 (149-164) | 150 (136-166) | | NA | NA | NA | NA |
| P (mg/dl) | 5.4 (2.9-10.6) | 6.8 (3.7-11.2) | 6.5 ± 1.2 (4.0-8.1) | 8.1 (3.8-15.8) | 9.3 (5.7-16.8) | 7.4 (3.3-13.4) | | 8.02 ± 2.79 (0.37-12.01)^f^ | NA | NA | NA |
| pCO_2_ (mmHg) | 36.1 (14.2-61.0) | NA | NA | NA | NA | NA | | NA | NA | NA | NA |
| PCV/Hct (%) | 37 (28-68) | 31 (9-40) | 32 ± 5 (18-40) | NA | 21 (14-32) | NA | | NA | 23 ± 6 (13-30) | 29 ± 5 (21-33) | NA |
| pH | 7.515 (7.208-7.925) | NA | NA | NA | NA | NA | | NA | NA | NA | NA |
| Plasma Protein (g/dl) | 4.2 (2.5-33.2) | NA | NA | NA | NA | NA | | NA | NA | NA | NA |
| pO_2_ (mmHg) | 68 (39-104) | NA | NA | NA | NA | NA | | NA | NA | NA | 60 |
| TCO_2_ (mmol/L) | 39.0 (21.7-52.7) | NA | NA | NA | NA | NA | | NA | NA | NA | NA |
| Total Protein (g/dl) | 3.9 (2.4-5.9) | 3.5 (2.1-6.0) | 3.7 ± 1.1 (1.6-5.6) | 4.1 (2.0-6.9) | 3.7 (2.3-4.7) | 3.0 (2.1-4.0) | | 4.28 ± 0.84 (1.40-5.97)^h^ | NA | NA | NA |
| Total Solids | 5.0 (3.0-7.4) | NA | NA | NA | NA | NA | | NA | NA | NA | NA |
| Uric Acid (mg/dl) | 1.3 (0-3.3) | 0.8 (0.1-2.8) | 0.7 (0.2-1.2) | 0.8 (0.1-2.3) | 0.8 (0.5-2.5) | 1.3 (1.0-2.4) | | NA | NA | NA | NA |
| WBC(count) (THOUS.) | 11.7 (3.5-15.0) | 9.0 (2.0-27.0) | NA | NA | NA | NA | | NA | 23.82 ± 11.28 (12-45)^i^ | 22.80 ± 11.03 (13-39) ^i^ | NA |
| WBC(max) (THOUS.) | 12.7 (4.5-16.0) | NA | NA | NA | NA | NA | | NA | NA | NA | NA |
| WBC(min) (THOUS.) | 10.7 (2.5-14.0) | NA | NA | NA | NA | NA | | NA | NA | NA | NA |
| ^a^ Taken from Stacy *et al*. (2018) calculations based on CCL to SCL conversion from Bjorndal *et al.* (2000)  ^b^ SJ – small juvenile, J – juvenile, LJ – large juvenile, SA – subadult, A – adult; based on age size classifications from Turtle Expert Working Group (2009)  ^c^ BUN values from converted from mmol/L to mg/dl to facilitate comparison to other studies  ^d^ Creatinine values were converted from µmol/L to mg/dl to facilitate comparison  ^e^ Albumin values were calculated from two protein fractions of albumin and converted from g/l to g/dl  ^f^ Values for Ca, Cholesterol, Glucose, P were converted from mmol/L to mg/dl to facilitate comparison to present study  ^g^ Globulin values were calculated from three protein fractions of alpha, beta, and gamma globulins and converted from g/l to g/dl  ^h^ Total Protein values were converted from g/l to g/dl  ^i^ WBC values were converted from µL^-3^ to THOUS. count for facilitated conversion | | | | | | |  |  |  |  |  |
|  | | | | | | |  |  |  |  |  |
